# Socioeconomic inequalities in HRQoL in England: an age-sex stratified analysis

**DOI:** 10.1186/s12955-022-02024-7

**Published:** 2022-08-02

**Authors:** Paul Schneider, James Love-Koh, Simon McNamara, Tim Doran, Nils Gutacker

**Affiliations:** 1 School of Health and Related Research, University of Sheffield, Sheffield, UK; 2 Centre for Health Economics, University of York, York, UK; 3 Lumanity, Sheffield, UK; 4 Department of Health Sciences, University of York, York, UK

**Keywords:** Health inequality, Health-related quality of life, EQ-5D, United Kingdom

## Abstract

**Background:**

Socioeconomic status is a key predictor of lifetime health: poorer people can expect to live shorter lives with lower average health-related quality-of-life (HRQoL) than richer people. In this study, we aimed to improve understanding of the socioeconomic gradient in HRQoL by exploring how inequalities in different dimensions of HRQoL differ by age.

**Methods:**

Data were derived from the Health Survey for England for 2017 and 2018 (14,412 participants). HRQoL was measured using the EQ-5D-5L instrument. We estimated mean EQ-5D utility scores and reported problems on five HRQoL dimensions (mobility, self-care, usual activities, pain/discomfort, anxiety/depression) for ages 16 to 90+ and stratified by neighbourhood deprivation quintiles. Relative and absolute measures of inequality were assessed.

**Results:**

Mean EQ-5D utility scores declined with age and followed a socioeconomic gradient, with the lowest scores in the most deprived areas. Gaps between the most and least deprived quintiles emerged around the age of 35, reached their greatest extent at age 60 to 64 (relative HRQoL of most deprived compared to least deprived quintile: females = 0.77 (95% CI: 0.68–0.85); males = 0.78 (95% CI: 0.69–0.87)) before closing again in older age groups. Gaps were apparent for all five EQ-5D dimensions but were greatest for mobility and self-care.

**Conclusion:**

There are stark socioeconomic inequalities in all dimensions of HRQoL in England. These inequalities start to develop from early adulthood and increase with age but reduce again around retirement age.

**Supplementary Information:**

The online version contains supplementary material available at 10.1186/s12955-022-02024-7.

## Introduction

Socioeconomic status is an important determinant of lifetime health [1, 2]. Individuals with lower educational attainment, occupational status, household income or those living in more socioeconomically deprived neighbourhoods live, on average, shorter lives than more advantaged individuals [3–5]. They also typically experience more and more complex health problems throughout their lifetime[6, 7] with negative consequences for their health-related quality-of-life (HRQoL) [8]. These inequalities in mortality and morbidity combine to generate significant variations in healthy life expectancy, which are widely perceived to be unfair.

Socioeconomic inequalities in health outcomes develop from the age of 20 for chronic morbidity [7] and age of 30 for mortality [9]. These gaps become more pronounced with increasing age, before receding in the oldest age groups as the limits of life are approached. Wider socioeconomic gaps, emerging earlier in life, are often evident for single-item measures of self-rated health [10], which nevertheless demonstrate similar patterns to physician diagnosed morbidity and mortality, with gaps increasing with age before narrowing in later life. This suggests that self-rated health may be more sensitive to the effects of acute illness and the early development of longer-term conditions, but accurate assessment is challenging because many measures of self-rated health conflate multiple dimensions of health and are subject to reporting bias, particularly for older people with multiple conditions [11].

HRQoL is a multidimensional concept and reflects physical, mental and social well-being. Monitoring of socioeconomic inequalities in HRQoL has only recently become possible through large-scale patient or population surveys that include suitable instruments such as the EQ-5D-5L to capture more nuanced aspects of HRQoL. Inequalities in HRQoL have been widely reported for whole populations with, on average, worse HRQoL reported in more deprived communities [12–17]. However, little is known about how socioeconomic inequalities in HRQoL differ with age and in which age groups inequalities in HRQoL are most pronounced [18]. The aim of this study is to investigate this question and provide estimates of socioeconomic inequalities in HRQoL across age groups in England.

## Methods

### Data

We used data from the 2017 and 2018 Health Survey for England (HSE), a long-running annual survey of a random sample of the English population, including children and adolescents [19, 20]. Participants were asked to report their health state using the EQ-5D-5L instrument. The EQ-5D-5L is a standardised patient-reported measure of HRQoL designed and validated for use in population health surveys [21]. HRQoL is assessed along five dimensions: mobility, self-care, usual activities, pain/discomfort and anxiety/depression. For each dimension, survey participants indicated the level of problems they were experiencing at the time of the survey on a five-point scale, ranging from no problems to extreme problems. The resulting responses form a health profile, which are summarised using preference estimates of the UK general population [22]. Summary scores range from 1 (indicating full health) to -0.6, with 0 being equivalent to being dead and scores below 0 indicating health states considered worse than being dead.

The HSE also provided information on patients’ age at the time of the survey (in 5-year age bands, with separate groups for 16–17, 18–19 and 90 + years of age), sex and an indicator of socioeconomic deprivation. The latter was measured using the Index of Multiple Deprivation (IMD), which combines multiple dimensions of relative deprivation (e.g. employment, income, education and housing, among other aspects) into a single deprivation score [23]. The IMD is defined at the level of Lower-Layer Super Output Areas (LSOAs), small geographical areas containing a median of around 1500 residents. Participants were linked to LSOAs based on their postcode of residence and were assigned to one of five socioeconomic groups (from 1, the most deprived, to 5, the least deprived) based on IMD quintiles for all LSOAs in England.

The HSE does not collect EQ-5D-5L responses for children and adolescents younger than 16 years. We therefore restricted our analysis to participants aged 16 or over at the time of the survey.

### Analysis

We assessed the mean HRQoL at different age groups (between 16 and 85+), stratified by IMD quintile. Socioeconomic inequalities in HRQoL for each age-sex group were assessed using three measures of inequality. The absolute difference is the difference in mean EQ-5D-5L index score between the most and least deprived quintiles. The relative difference is the ratio of most over least deprived quintiles, with a value of 1 indicating equal levels of HRQoL (no inequality) and values below 1 indicating lower levels of HRQoL in more deprived populations. Both these measures are easily interpretable but do not reflect inequalities among the second to fourth quintile. We therefore also present concentration indices, which rank quintiles from most to least deprived and plots the cumulative proportion of health against the cumulative proportion of the population. The observed distribution of HRQoL is then compared with a hypothetical equal distribution. The index ranges from − 1 to 1, with these extreme values reflecting scenarios in which the most deprived or least deprived quintile, respectively, hold all of the health variable. A value of 0 represents perfect equality. The 95% confidence intervals around each of these statistics were derived by bootstrapping using 1000 iterations.

Furthermore, we investigated the inequality within the five EQ-5D-5L dimensions by plotting the proportion of participants in each age group and IMD strata reporting no, slight, moderate, severe, and extreme problems. Relative differences were computed by comparing the proportion of individuals reporting no problems across the most and least deprived deprivation quintiles.

All analyses were conducted separately for males and females. HSE survey weights were applied to adjust for non-contact and refusal of households as well as for individual non-response within households to generate a representative sample of the English population with respect to age and sex.

Statistical analyses were conducted in R version 4.1.0 or later. No ethics approval was required for analysis of anonymised secondary data.

## Results

A total of 16,175 participants aged 16 or over took part in the HSE waves of 2017 and 2018. Of these, 1763 (10.9%) were excluded because they provided incomplete EQ-5D-5L responses, leaving a final sample of 14,412 participants for analysis (n = 7168 for 2017 and n = 7244 for 2018). See Additional file 1: Table S1 for counts of excluded observations by age, sex and deprivation group.

Table 1 presents descriptive statistics of the analysis sample (weighted) and Additional file 1: Fig. S1 in the online supplementary material plots the distribution of EQ-5D-5L summary scores. HRQoL decreased with increasing age, from mean 0.923 for the 16–19 age group to 0.702 for ages 85+ for males, and from 0.873 to 0.665 for females. For all age groups women reported lower mean HRQoL than men. Participants who lived in deprived areas had, on average, lower EQ-5D indices than those who lived in less deprived areas.

**Table 1:** Descriptive statistics of the analysis sample, weighted using HSE survey weights

	N (%)	EQ-5D-5L	N (%)	EQ-5D-5L	N (%)	EQ-5D-5L
		Mean (SD)		Mean (SD)		Mean (SD)
*Age*	Total (weighted)		Female (weighted)		Male (weighted)	
16–19	815.6 (5.7%)	0.899 (0.148)	387.8 (5.2%)	0.873 (0.171)	427.8 (6.2%)	0.923 (0.118)
20–24	1014.8 (7.1%)	0.880 (0.172)	523.6 (7.0%)	0.866 (0.184)	491.2 (7.1%)	0.895 (0.156)
25–29	1144.0 (8.0%)	0.884 (0.177)	561.0 (7.6%)	0.873 (0.188)	583.0 (8.4%)	0.895 (0.165)
30–34	1287.5 (9.0%)	0.891 (0.163)	684.8 (9.2%)	0.870 (0.179)	602.7 (8.7%)	0.916 (0.138)
35–39	1129.6 (7.9%)	0.859 (0.203)	594.4 (8.0%)	0.857 (0.189)	535.2 (7.7%)	0.862 (0.218)
40–44	1168.5 (8.1%)	0.860 (0.204)	593.3 (8.0%)	0.850 (0.211)	575.2 (8.3%)	0.870 (0.196)
45–49	1240.6 (8.6%)	0.819 (0.248)	634.7 (8.5%)	0.815 (0.239)	605.9 (8.7%)	0.824 (0.256)
50–54	1298.6 (9.0%)	0.821 (0.232)	661.6 (8.9%)	0.805 (0.251)	637.0 (9.2%)	0.837 (0.210)
55–59	1175.2 (8.2%)	0.809 (0.245)	627.8 (8.5%)	0.802 (0.256)	547.4 (7.9%)	0.817 (0.231)
60–64	971.5 (6.8%)	0.797 (0.246)	481.5 (6.5%)	0.784 (0.245)	490.0 (7.1%)	0.809 (0.246)
65–69	927.5 (6.5%)	0.790 (0.242)	477.3 (6.4%)	0.782 (0.245)	450.2 (6.5%)	0.798 (0.239)
70–74	867.2 (6.0%)	0.794 (0.211)	459.9 (6.2%)	0.787 (0.213)	407.3 (5.9%)	0.802 (0.209)
75–79	594.9 (4.1%)	0.763 (0.228)	335.6 (4.5%)	0.741 (0.234)	259.3 (3.7%)	0.791 (0.217)
80–84	405.3 (2.8%)	0.742 (0.235)	226.1 (3.0%)	0.717 (0.233)	179.2 (2.6%)	0.773 (0.234)
85 +	311.2 (2.2%)	0.681 (0.255)	177.7 (2.4%)	0.665 (0.244)	133.5 (1.9%)	0.702 (0.269)
*IMD quintile*						
Q5 (Least deprived)	2766.8 (19.3%)	0.863 (0.177)	1443.8 (19.4%)	0.857 (0.177)	1323.1 (19.1%)	0.871 (0.176)
Q4	3018.1 (21.0%)	0.850 (0.197)	1523.4 (20.5%)	0.834 (0.208)	1494.8 (21.6%)	0.865 (0.184)
Q3	2975.6 (20.7%)	0.842 (0.205)	1517.6 (20.4%)	0.827 (0.208)	1458.1 (21.1%)	0.857 (0.200)
Q2	2990.5 (20.8%)	0.812 (0.240)	1572.3 (21.2%)	0.801 (0.237)	1418.2 (20.5%)	0.824 (0.243)
Q1 (Most deprived)	2601.0 (18.1%)	0.796 (0.262)	1370.1 (18.4%)	0.777 (0.275)	1230.8 (17.8%)	0.818 (0.245)
Total	14,352.1 (100%)	0.833 (0.2189)	7427.1 (51.7%)	0.819 (0.225)	6925.0 (48.3%)	0.848 (0.212)

### Inequality in HRQoL

**Fig. 1:**
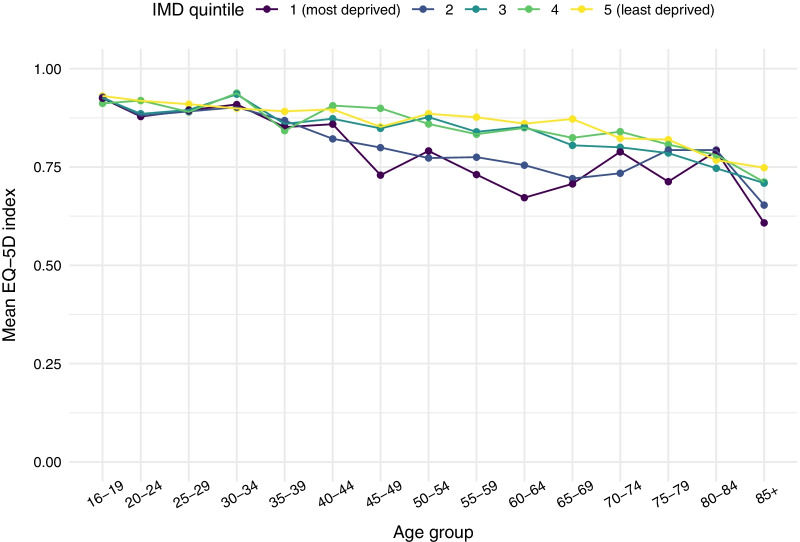
Mean EQ-5D score by IMD quintile and age group—males

**Fig. 2:**
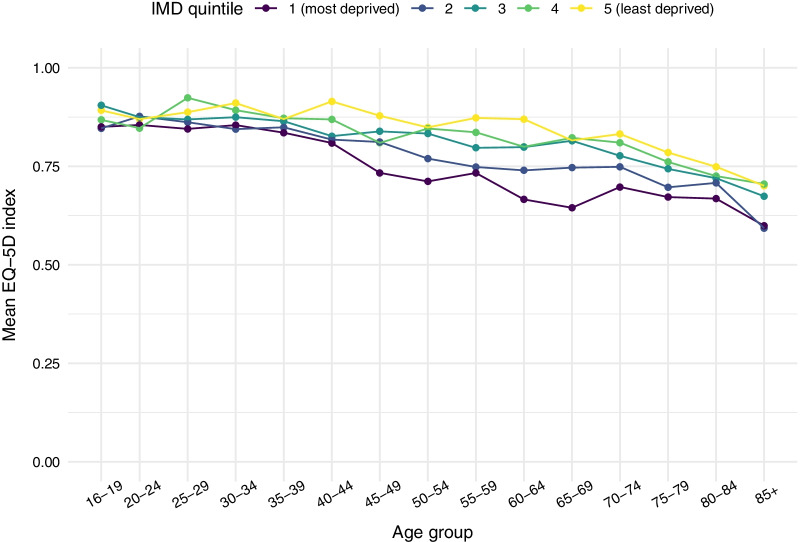
Mean EQ-5D score by IMD quintile and age group—females

Figures 1 and 2 show mean EQ-5D-5L utility scores by age and IMD quintile, for males and females respectively. Socioeconomic gaps in HRQoL were small in younger age groups, but started to widen around the age of 40, reaching their maximum at approximately 60–64. As people enter retirement age, the absolute gap in HRQoL begins to close again. Further details on mean HRQoL values and 95% confidence intervals by age and IMD quintile are provided in Additional file 1: Tables S2 and S3.

**Fig. 3:**
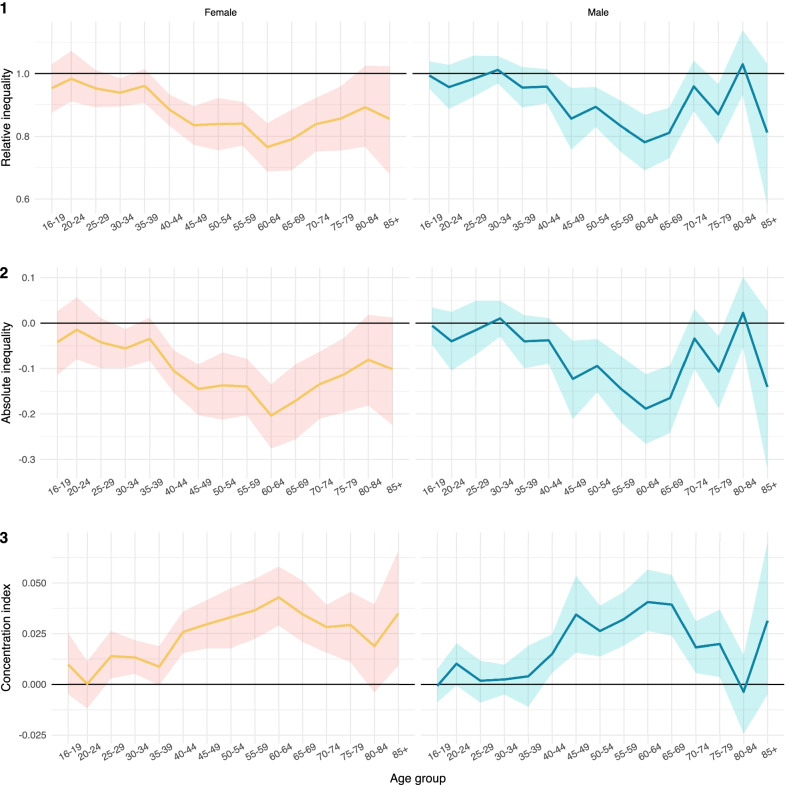
Measures of inequality in EQ-5D-5L utility scores by age and sex

Panel 1 of Fig. 3 show the relative difference (i.e. the ratio) between the most deprived and least deprived groups with associated 95% confidence intervals, by age group and sex. Although the ratio is nearly always estimated to be lower than one for both sexes, up to the 35–39 age group these estimated differences are not statistically significant (see also Additional file 1: Tables S2 and S3), suggesting limited socioeconomic inequality in HRQoL at younger ages. The ratio then declines with increasing age, falling to minimum values of 0.77 (95%CI: 0.68–0.85) for females and 0.78 (95%CI: 0.69–0.87) for males in the 60–64 age group, respectively, before increasing again in the oldest age groups. In most age groups, inequality tended to be higher (i.e. the ratio was lower) in females, but these differences were not statistically significant. For males, the inequality ratio estimate becomes unstable in later age groups due to relatively small sample sizes.

Absolute differences in EQ-5D utility scores between the most and least deprived groups (Panel 2) are large and clinically relevant. McClure and colleagues [24] estimate that differences in EQ-5D-5L utility scores exceeding 0.063 are likely to be considered meaningful in our population (i.e. the minimally important difference (MID)). For females, absolute gaps between the most and least deprived groups exceed the MID between the ages of 40–74. For males, the age range is slightly shorter (45–69).

Panel 3 of Fig. 3 shows similar patterns of age-specific inequality using concentration indices. These indices are positive for nearly all age groups, indicating that a greater share of HRQoL is concentrated in lower deprivation quintiles. The concentration index scores increase with age before peaking for the 60–64 age group.

### Inequalities by EQ-5D-5L dimension

**Fig. 4:**
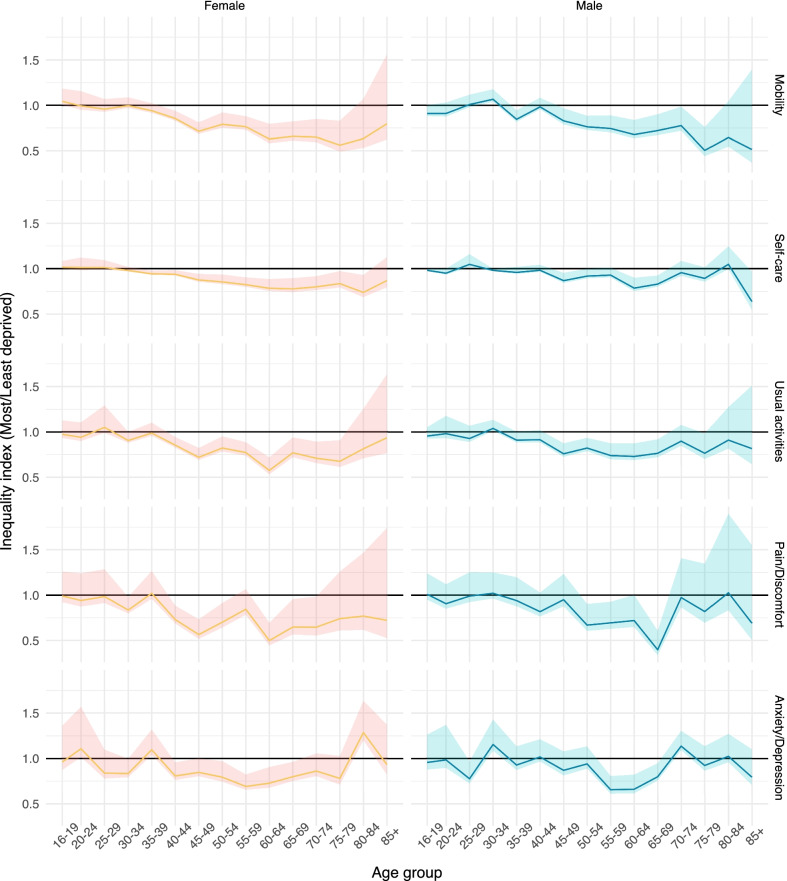
Relative differences in the proportion of respondents reporting no problems for each EQ-5D-5L dimension by age and sex

Figure 4 plots the relative difference in the proportions of those reporting slight problems or worse between IMD quintiles 1 (most deprived) and 5 (least deprived) by age and sex (see Additional file 1: Figs. S2 and S3 in the online supplementary material for the proportion of participants reporting no, slight, moderate, severe and extreme problems on each of the five EQ-5D-5L dimensions). The patterns for pain/discomfort, anxiety/depression and self-care are broadly similar, with inequalities emerging between ages 30 and 40, peaking between 55 and 65 and reducing thereafter. Inequalities in usual activities are consistent from age 45 onwards, whilst inequalities in mobility problems follow a different pattern, steadily increasing with age and peaking at a value of 0.5 for the 75–79 age group.

## Discussion

Our analysis, based on a multi-dimensional measure of health-related quality-of-life (HRQoL) applied across all adult age groups, illustrates the dynamic nature of socioeconomic inequalities in health in England. We found that HRQoL declined with increasing age across all five dimensions measured by the EQ-5D-5L. However, there were clear interactions between age and deprivation, with quality of life for people living in deprived areas in England declining substantially faster with age than those living in more advantaged areas. Our analysis shows that inequalities in overall HRQoL follow an approximately U-shape pattern: deprivation-related gaps emerge around the age of 35, reach a peak at around 60 to 64, and then decline again after retirement age. These patterns were consistent when using either the simple absolute or relative difference measures or the more complex concentration index that includes information on all deprivation quintiles. Inequalities in individual dimensions of HRQoL follow a similar pattern, except for mobility, where the gap continues to increase with age. Overall, our results provide further evidence that socioeconomic status is a key predictor of lifetime health in England.

### Strengths and limitations

The key strengths of our study are the representativeness of the HSE of the general population in England, the use of an established and widely accepted HRQoL measure such as the EQ-5D-5L, the lower incidence of ceiling effects compared to the three level version of the instrument (EQ-5D-3L) used in previous studies of the English population [15, 16], and the large sample size which permits stratifying HRQoL analyses by age, sex and area-based measures of deprivation.

However, there are also limitations to the study. First, we use the two most recent waves of the HSE, which offer an accurate picture of the current levels of inequality in HRQoL by age and sex but may be subject to cohort effect that limit the ability to extrapolate to past or future cohorts e.g. when calculating quality-adjusted life expectancy [15, 25, 26]. However, a previous study examining changes in EQ-5D-5L responses in England found that scores were stable for most domains and age groups between 2012 and 2017, with the exception of scores for anxiety/depression, which deteriorated for the under 35 s and for women, with the greatest change (leading to a 1.3% decrease in overall HRQoL) in the fifth of women in the most deprived areas [17].

Second, approximately 11% of participants did not report their HRQoL and were excluded from the study. Men and those living in deprived neighbourhoods were more likely to have missing HRQoL information, which may have affected our analysis. We did not impute missing values given that Love-Koh et al. [15] found imputation to have at best a marginal impact on HRQoL scores by deprivation quintile group: mean estimates of HRQoL between naive and imputed datasets differed by less than 0.01.

Third, our finding of inequality in mean EQ-5D-5L index scores (but not in dimension responses) is contingent on the value set chosen to derive these scores. A UK valuation study for the EQ-5D-5L is currently underway and this may affect the results presented here [27].

Fourth, our results need to be interpreted in the context of differential life expectancy across socio-economic deprivation groups, with people in more deprived neighbourhoods in England expected to live significantly shorter lives [28]. Dead people have a defined EQ-5D utility score of zero but are not included in the HSE. As a result, our study measures inequality in HRQoL conditional on being alive.

Finally, it is possible that individuals of different socioeconomic backgrounds report the same level of HRQoL differently on the EQ-5D instrument. For example, individuals may differ in how they interpret limitations of their ‘usual activities’ according to occupation, education or lifestyle [29, 30]. More research is needed to quantify such reporting heterogeneity and adjust for it in the calculation of inequalities in HRQoL.

### Findings

As expected, we found that HRQoL varies with age, sex and deprivation. With respect to age, mean EQ-5D-5L utility scores start to decline from age 45–49 onwards, and this pattern is also found in three of the HRQoL dimensions—mobility, self-care and usual activities—for which there is generally low prevalence of problems in younger people. There are similar increases for pain/discomfort from middle age, but in this case a significant minority of younger people also report at least slight problems with pain. There is a different pattern for anxiety/depression, with around a quarter of respondents reporting problems in every age group.

Previous survey studies using the EQ-5D have found inequalities in all domains, with the greatest gaps for the pain and anxiety/depression domains [16]. Linked studies have also shown that obesity and chronic conditions, particularly stroke and mental illness, are strong predictors of lower HRQoL scores, but these impacts are mitigated to some extent by higher social status [18]. Shah et al. [17] examining responses to the national GP Patient Survey in England, found lower average overall HRQoL scores for women and a steep deterioration with age. Previous studies have not, however, measured age-related inequalities by domain. In this study, we also found a clear socioeconomic gradient in mean overall EQ-5D-5L scores, with increasing deprivation associated with lower HRQoL, but this gradient only started to emerge in middle age, after which age-related declines in HRQoL were much steeper in more deprived areas.

This interaction between age and deprivation leads to some striking inequalities in HRQoL; for example, in 2017–2018, average HRQoL was lower for 45–49 year old males living in the most deprived fifth of neighbourhoods than for 75–79 year old males living in the least deprived fifth. For four of the five dimensions of the EQ-5D-5L socioeconomic inequalities followed a U-shaped distribution, increasing between ages 45–49 and 60–64 and then decreasing in the older age groups. This was due to declines in HRQoL emerging in younger age groups in more deprived areas, with rates of reported problems eventually converging on similar levels across all quintiles in the oldest age groups. This convergence may in part reflect a healthy survivor effect, which would be consistent with prior evidence on the interaction of age, deprivation and multi-morbidity [7]. Despite inequalities in HRQoL not becoming apparent before middle age, it is likely that they reflect socioeconomic conditions present from childhood and the prenatal period that impact health in ways that may not be detectable by EQ-5D-5L (for example, low birthweight and obesity), but which compromise adult health in the long term [31]. These socially determined shortfalls in health may then be compounded by deprived groups receiving less health care than more advantaged groups, relative to their additional needs [32].

For mobility, inequalities continued to increase with age, with respondents in less deprived areas never reaching the same average levels of problems with walking as those in more deprived areas, even in the oldest age groups. This was particularly apparent in the proportion reporting severe or extreme problems, which ranged from 17% for women and 14% for men in the least deprived fifth of neighbourhoods to 27% for women and 20% for men in the most deprived fifth for the 85+ age group. In addition to differences in prevalence and severity of conditions associated with restricted mobility, this may also reflect differences in daily activities; the social and physical environment; and access to appropriate assistance, mobility aids and means of transport to mitigate the impacts of reduced mobility [33]. Age and deprivation-related patterns in HRQoL, and the interactions between them, were very similar for both sexes, with women tending to report more problems for all ages and all levels of deprivation across all domains.

### Conclusion

Our findings highlight the need to use a range of measures in additions to life expectancy and summary measures of morbidity when monitoring health inequalities. Quality-adjusted life expectancy has been proposed [25, 26] as a metric that combines life expectancy with information on HRQoL to capture the cumulative impact of morbidity over individuals’ life spans. However, measuring mean differences in HRQoL across socioeconomic groups conditional on age as done in previous studies [15, 16] may fail to account for differences in inequalities in HRQoL at different points of the life course. We found that inequalities in HRQoL between the most and least deprived socioeconomic groups change with age, following an approximately U-shaped pattern for most dimensions. These results could be used to refine QALE estimates by accounting explicitly for changes in HRQoL inequalities over the life cycle. Future research could also explore inequalities by age for other equity-sensitive characteristics such as ethnicity [34].

## Supplementary Information

**Additional file 1:** Online supplementary material to ‘Socioeconomic inequalities in HRQoL in England: an age-sex stratified analysis’.

## Data Availability

The datasets generated and/or analysed during the current study are available in the UK Data Archive at Essex University, https://doi.org/10.5255/UKDA-SN-8860-1 and from the Office for National Statistics, URL: https://www.ons.gov.uk/peoplepopulationandcommunity/healthandsocialcare/healthinequalities/bulletins/healthstatelifeexpectanciesbyindexofmultipledeprivationimd/2017to2019
