# Supplementary material for: Socioeconomic inequalities in HRQoL in England: an age-sex stratified analysis
Source: Health Qual Life Outcomes. 2022 Aug 2;20:121. doi: 10.1186/s12955-022-02024-7 (PMC9347153; doi:10.1186/s12955-022-02024-7)
Supplement: Supplementary file 1 — Additional file 1: Online supplementary material to ‘Socioeconomic inequalities in HRQoL in England: an age-sex stratified analysis’. [file 12955_2022_2024_MOESM1_ESM.docx]

Online supplementary material to ‘Socioeconomic inequalities in HRQoL in England: an age-sex stratified analysis’


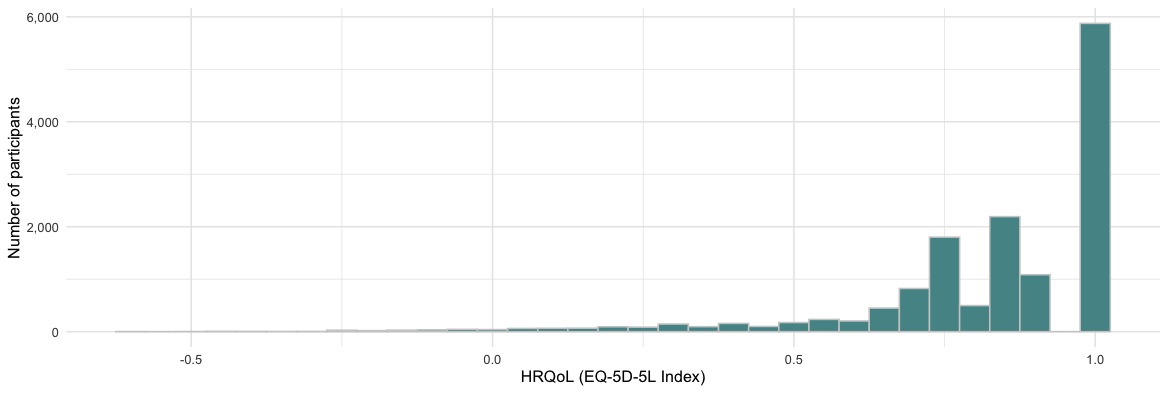


Figure S1: Distribution of EQ-5D-5L summary scores in the analysis sample


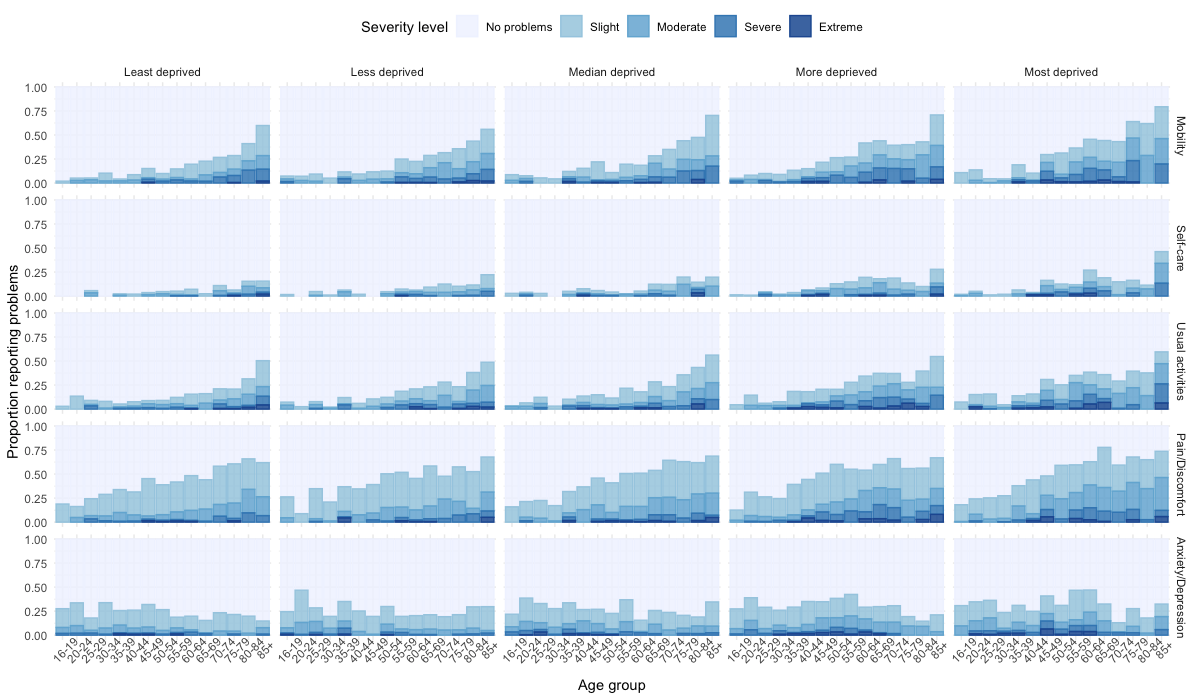


Figure S2. Proportion reporting each level of response for each EQ-5D dimension over age - Males


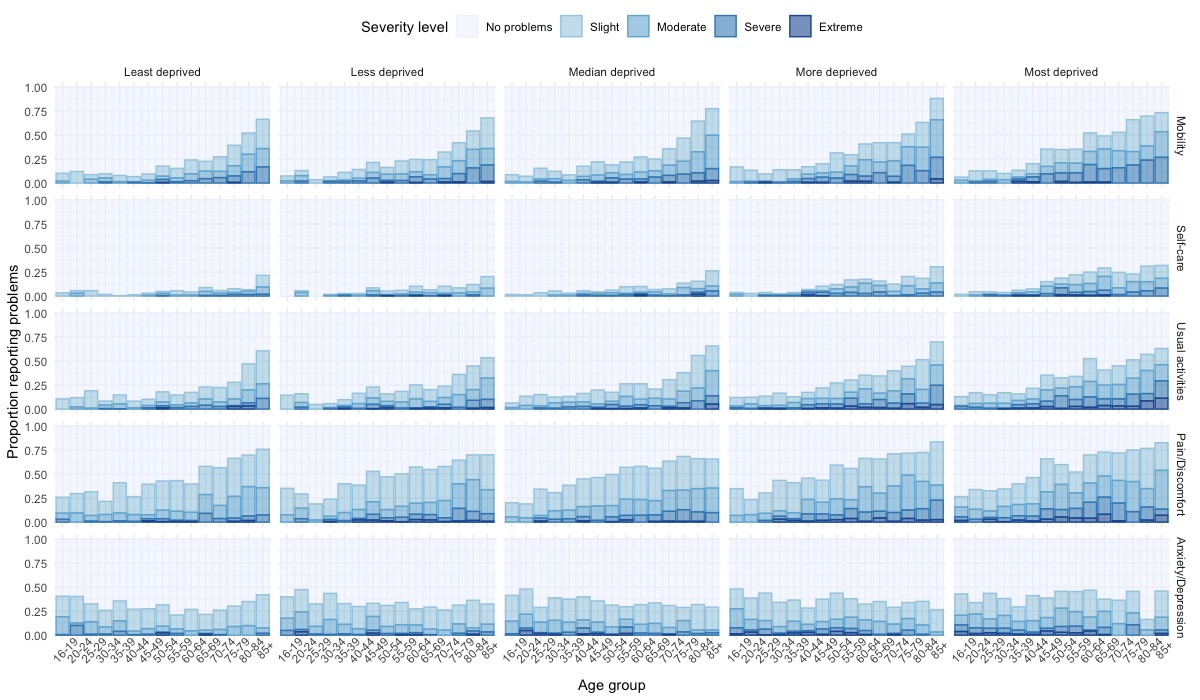


Figure S3. Proportion reporting each level of response for each EQ-5D dimension over age - Females

Table S1: Counts of observation with missing EQ-5D-5L data by age group, sex and IMD quintile

|  | N (%) |
| --- | --- |
| **By sex** |  |
| Female | 876 (9.8%) |
| Male | 886 (12.3%) |
|  |  |
| **By IMD quintile** |  |
| 5 (least deprived) | 302 (9.5%) |
| 4 | 341 (9.9%) |
| 3 | 385 (11.8%) |
| 2 | 337 (10.4%) |
| 1 (most deprived) | 397 (13.1%) |
|  |  |
| **By Age** |  |
| 16-19 | 92 (14.8%) |
| 20-24 | 106 (13.7%) |
| 25-29 | 108 (10.9%) |
| 30-34 | 114 (9.2%) |
| 35-39 | 121 (9.3%) |
| 40-44 | 151 (11.5%) |
| 45-49 | 129 (9.6%) |
| 50-54 | 133 (9.6%) |
| 55-59 | 131 (9.1%) |
| 60-64 | 126 (10%) |
| 65-69 | 115 (8.8%) |
| 70-74 | 138 (10.9%) |
| 75-79 | 128 (14.8%) |
| 80-84 | 73 (12.7%) |
| 85+ | 97 (20%) |

Table S2: Mean (95% confidence interval) EQ-5D-5L score by age group and IMD - males

| **Age** | **1 (least deprived)** | **2** | **3** | **4** | **5 (most deprived)** | **Ratio Q5/Q1** | **Difference Q5-Q1** | **Concentration index** |
| --- | --- | --- | --- | --- | --- | --- | --- | --- |
| **16-20** | 0.93 (0.51; 1.35) | 0.91 (0.49; 1.33) | 0.93 (0.51; 1.35) | 0.93 (0.51; 1.35) | 0.92 (0.50; 1.34) | 0.99 (0.95; 1.04) | -0.01 (-0.05; 0.03) | -0.00 (-0.01 ;0.01) |
| **20-24** | 0.92 (0.50; 1.34) | 0.92 (0.50; 1.34) | 0.89 (0.47; 1.30) | 0.88 (0.46; 1.30) | 0.88 (0.46; 1.30) | 0.96 (0.89; 1.03) | -0.04 (-0.10; 0.02) | 0.01 (-0.00 ;0.02) |
| **25-29** | 0.91 (0.49; 1.33) | 0.89 (0.47; 1.31) | 0.90 (0.48; 1.31) | 0.89 (0.47; 1.31) | 0.89 (0.48; 1.31) | 0.98 (0.93; 1.06) | -0.02 (-0.07; 0.05) | 0.00 (-0.01 ;0.01) |
| **30-34** | 0.90 (0.48; 1.32) | 0.94 (0.52; 1.36) | 0.93 (0.52; 1.35) | 0.90 (0.48; 1.32) | 0.91 (0.49; 1.33) | 1.01 (0.97; 1.06) | 0.01 (-0.03; 0.05) | 0.00 (-0.01 ;0.01) |
| **35-39** | 0.89 (0.47; 1.31) | 0.84 (0.42; 1.26) | 0.86 (0.44; 1.28) | 0.87 (0.45; 1.29) | 0.85 (0.43; 1.27) | 0.95 (0.89; 1.02) | -0.04 (-0.10; 0.02) | 0.00 (-0.01 ;0.02) |
| **40-44** | 0.90 (0.48; 1.32) | 0.91 (0.49; 1.33) | 0.87 (0.45; 1.29) | 0.82 (0.40; 1.24) | 0.86 (0.44; 1.28) | 0.96 (0.90; 1.01) | -0.04 (-0.09; 0.01) | 0.01 (0.01 ;0.02) |
| **45-49** | 0.85 (0.43; 1.27) | 0.90 (0.48; 1.32) | 0.85 (0.43; 1.27) | 0.80 (0.38; 1.22) | 0.73 (0.31; 1.15) | 0.86 (0.76; 0.95) | -0.12 (-0.21; -0.04) | 0.03 (0.02 ;0.05) |
| **50-54** | 0.89 (0.47; 1.30) | 0.86 (0.44; 1.28) | 0.88 (0.46; 1.30) | 0.77 (0.35; 1.19) | 0.79 (0.37; 1.21) | 0.89 (0.83; 0.96) | -0.09 (-0.15; -0.04) | 0.03 (0.01 ;0.04) |
| **55-59** | 0.88 (0.46; 1.30) | 0.83 (0.41; 1.25) | 0.84 (0.42; 1.26) | 0.78 (0.36; 1.19) | 0.73 (0.31; 1.15) | 0.83 (0.75; 0.91) | -0.15 (-0.22; -0.07) | 0.03 (0.02 ;0.05) |
| **60-64** | 0.86 (0.44; 1.28) | 0.85 (0.43; 1.27) | 0.85 (0.43; 1.27) | 0.75 (0.34; 1.17) | 0.67 (0.25; 1.09) | 0.78 (0.69; 0.87) | -0.19 (-0.27; -0.11) | 0.04 (0.03 ;0.06) |
| **65-69** | 0.87 (0.45; 1.29) | 0.82 (0.41; 1.24) | 0.81 (0.39; 1.22) | 0.72 (0.30; 1.14) | 0.71 (0.29; 1.13) | 0.81 (0.73; 0.89) | -0.16 (-0.24; -0.09) | 0.04 (0.02 ;0.05) |
| **70-74** | 0.82 (0.40; 1.24) | 0.84 (0.42; 1.26) | 0.80 (0.38; 1.22) | 0.73 (0.31; 1.15) | 0.79 (0.37; 1.21) | 0.96 (0.88; 1.04) | -0.03 (-0.10; 0.03) | 0.02 (0.01 ;0.03) |
| **75-79** | 0.82 (0.40; 1.24) | 0.81 (0.39; 1.23) | 0.79 (0.36; 1.21) | 0.79 (0.37; 1.22) | 0.71 (0.29; 1.14) | 0.87 (0.77; 0.97) | -0.11 (-0.19; -0.03) | 0.02 (0.00 ;0.04) |
| **80-84** | 0.77 (0.35; 1.19) | 0.78 (0.36; 1.20) | 0.75 (0.32; 1.17) | 0.79 (0.37; 1.22) | 0.79 (0.36; 1.22) | 1.03 (0.93; 1.14) | 0.02 (-0.05; 0.10) | -0.00 (-0.02 ;0.01) |
| **85+** | 0.75 (0.32; 1.17) | 0.71 (0.29; 1.14) | 0.71 (0.29; 1.13) | 0.65 (0.23; 1.08) | 0.61 (0.17; 1.04) | 0.81 (0.58; 1.03) | -0.14 (-0.32; 0.03) | 0.03 (-0.01 ;0.07) |

Table S3: Mean (95% confidence interval) EQ-5D-5L score by age group and IMD - females

| **Age** | **1 (least deprived)** | **2** | **3** | **4** | **5 (most deprived)** | **Ratio Q5/Q1** | **Difference Q5-Q1** | **Concentration index** |
| --- | --- | --- | --- | --- | --- | --- | --- | --- |
| **16-20** | 0.89 (0.48; 1.30) | 0.87 (0.46; 1.28) | 0.90 (0.50; 1.31) | 0.85 (0.44; 1.26) | 0.85 (0.44; 1.26) | 0.95 (0.87; 1.03) | -0.04 (-0.11; 0.03) | -0.00 (-0.01 ;0.01) |
| **20-24** | 0.87 (0.46; 1.28) | 0.85 (0.44; 1.26) | 0.87 (0.47; 1.28) | 0.88 (0.47; 1.28) | 0.85 (0.45; 1.26) | 0.98 (0.91; 1.07) | -0.01 (-0.08; 0.06) | 0.01 (-0.00 ;0.02) |
| **25-29** | 0.89 (0.48; 1.30) | 0.92 (0.52; 1.33) | 0.87 (0.46; 1.28) | 0.86 (0.45; 1.27) | 0.85 (0.44; 1.25) | 0.95 (0.89; 1.01) | -0.04 (-0.10; 0.01) | 0.00 (-0.01 ;0.01) |
| **30-34** | 0.91 (0.50; 1.32) | 0.89 (0.48; 1.30) | 0.87 (0.47; 1.28) | 0.84 (0.44; 1.25) | 0.85 (0.45; 1.26) | 0.94 (0.89; 0.98) | -0.06 (-0.10; -0.01) | 0.00 (-0.01 ;0.01) |
| **35-39** | 0.87 (0.46; 1.28) | 0.87 (0.46; 1.28) | 0.86 (0.46; 1.27) | 0.85 (0.44; 1.26) | 0.84 (0.43; 1.24) | 0.96 (0.91; 1.01) | -0.03 (-0.08; 0.01) | 0.00 (-0.01 ;0.02) |
| **40-44** | 0.91 (0.51; 1.32) | 0.87 (0.46; 1.28) | 0.83 (0.42; 1.23) | 0.82 (0.41; 1.23) | 0.81 (0.40; 1.22) | 0.88 (0.83; 0.93) | -0.11 (-0.15; -0.06) | 0.01 (0.01 ;0.02) |
| **45-49** | 0.88 (0.47; 1.29) | 0.81 (0.40; 1.22) | 0.84 (0.43; 1.25) | 0.81 (0.40; 1.22) | 0.73 (0.33; 1.14) | 0.83 (0.77; 0.90) | -0.15 (-0.20; -0.09) | 0.03 (0.02 ;0.05) |
| **50-54** | 0.85 (0.44; 1.26) | 0.85 (0.44; 1.25) | 0.83 (0.43; 1.24) | 0.77 (0.36; 1.18) | 0.71 (0.30; 1.12) | 0.84 (0.76; 0.92) | -0.14 (-0.21; -0.06) | 0.03 (0.01 ;0.04) |
| **55-59** | 0.87 (0.47; 1.28) | 0.84 (0.43; 1.24) | 0.80 (0.39; 1.20) | 0.75 (0.34; 1.16) | 0.73 (0.33; 1.14) | 0.84 (0.77; 0.91) | -0.14 (-0.20; -0.08) | 0.03 (0.02 ;0.05) |
| **60-64** | 0.87 (0.46; 1.28) | 0.80 (0.39; 1.21) | 0.80 (0.39; 1.21) | 0.74 (0.33; 1.15) | 0.67 (0.26; 1.08) | 0.77 (0.69; 0.84) | -0.20 (-0.28; -0.13) | 0.04 (0.03 ;0.06) |
| **65-69** | 0.82 (0.41; 1.22) | 0.82 (0.41; 1.23) | 0.81 (0.41; 1.22) | 0.75 (0.34; 1.16) | 0.64 (0.24; 1.05) | 0.79 (0.69; 0.88) | -0.17 (-0.26; -0.09) | 0.04 (0.02 ;0.05) |
| **70-74** | 0.83 (0.42; 1.24) | 0.81 (0.40; 1.22) | 0.78 (0.37; 1.18) | 0.75 (0.34; 1.16) | 0.70 (0.29; 1.11) | 0.84 (0.75; 0.92) | -0.13 (-0.21; -0.06) | 0.02 (0.01 ;0.03) |
| **75-79** | 0.78 (0.38; 1.19) | 0.76 (0.35; 1.17) | 0.74 (0.33; 1.15) | 0.70 (0.29; 1.11) | 0.67 (0.26; 1.08) | 0.86 (0.75; 0.96) | -0.11 (-0.20; -0.03) | 0.02 (0.00 ;0.04) |
| **80-84** | 0.75 (0.34; 1.16) | 0.73 (0.31; 1.14) | 0.72 (0.31; 1.13) | 0.71 (0.30; 1.12) | 0.67 (0.26; 1.08) | 0.89 (0.77; 1.02) | -0.08 (-0.18; 0.02) | -0.00 (-0.02 ;0.01) |
| **85+** | 0.70 (0.29; 1.11) | 0.71 (0.29; 1.12) | 0.67 (0.26; 1.09) | 0.59 (0.18; 1.01) | 0.60 (0.19; 1.01) | 0.86 (0.68; 1.02) | -0.10 (-0.22; 0.01) | 0.03 (-0.01 ;0.07) |
